# Identification of MicroRNAs Inhibiting TGF-β-Induced IL-11 Production in Bone Metastatic Breast Cancer Cells

**DOI:** 10.1371/journal.pone.0037361

**Published:** 2012-05-21

**Authors:** Sirkku Pollari, Suvi-Katri Leivonen, Merja Perälä, Vidal Fey, Sanna-Maria Käkönen, Olli Kallioniemi

**Affiliations:** 1 Medical Biotechnology, VTT Technical Research Centre of Finland and Turku Centre for Biotechnology, University of Turku, Turku, Finland; 2 Institute of Biomedicine, University of Turku, Turku, Finland; 3 Institute for Molecular Medicine Finland (FIMM), University of Helsinki, Helsinki, Finland; University of Medicine and Dentistry of New Jersey, United States of America

## Abstract

Development of bone metastases is dependent on the cancer cell-bone cell interactions in the bone microenvironment. Transforming growth factor β (TGF-β) is released from bone during osteoclastic bone resorption and induces production of osteolytic factors, such as interleukin 11 (IL-11), in breast cancer cells. IL-11 in turn increases osteolysis by stimulating osteoclast function, launching a vicious cycle of cancer growth and bone destruction. We aimed to identify and functionally characterize microRNAs (miRNAs) that mediate the bone metastatic process, focusing on miRNAs that regulate the TGF-β induction of IL-11. First, we profiled the expression of 455 miRNAs in a highly bone metastatic MDA-MB-231(SA) variant as compared to the parental MDA-MB-231 breast cancer cell line and found 16 miRNAs (3.5%) having a >3-fold expression difference between the two cell types. We then applied a cell-based overexpression screen with Pre-miRNA constructs to functionally identify miRNAs regulating TGF-β-induced IL-11 production. This analysis pinpointed miR-204, miR-211, and miR-379 as such key regulators. These miRNAs were shown to directly target *IL11* by binding to its 3′ UTR. MiR-379 also inhibited Smad2/3/4-mediated transcriptional activity. Gene expression analysis of miR-204 and miR-379-transfected cells indicated that these miRNAs downregulated the expression of several genes involved in TGF-β signaling, including prostaglandin-endoperoxide synthase 2 (PTGS2). In addition, there was a significant correlation between the genes downregulated by miR-379 and a set of genes upregulated in basal subtype of breast cancer. Taken together, the functional evidence and clinical correlations imply novel mechanistic links between miRNAs and the key steps in the bone metastatic process in breast cancer, with potential clinical relevance.

## Introduction

Metastasis to bone is the most frequent cause of breast cancer morbidity and mortality. Currently available therapies are able to alleviate painful symptoms but bone metastatic cancer remains incurable. This is due to limited understanding of the integral molecular and cellular determinants of the bone metastatic process. Gene expression profiling of clinical tumor samples and experimental *in vivo* studies have revealed sets of genes whose expression in tumor cells correlates with their metastatic potential [1]. Many of these genes have been shown to play an important role in different phases of metastatic progression, but a therapeutically applicable common regulatory mechanism governing a multitude of these gene expression changes in tumor cells is yet to be discovered. MicroRNAs (miRNAs) are attractive candidates as multifunctional regulators of metastatic progression because one miRNA can regulate an entire set of genes. There is an increasing amount of evidence for under- and overexpression of several miRNAs in cancer, as compared to the normal tissue, and for the impact of miRNAs in epithelial-to-mesenchymal transition (EMT) [2] and metastatic progression (reviewed in [3]). The specific role of miRNAs in the bone metastatic process of breast cancer has not been extensively studied, but available early results suggest miRNAs as potential key regulators [4]–[6].

Transforming growth factor β (TGF-β) is one of the key tumor-promoting growth factors in advanced cancers. It induces EMT and has a key role in the bone metastatic process, in the vicious cycle between bone and breast cancer cells. TGF-β regulates cell type-specific transcriptional responses via canonical Smad and non-Smad signaling pathways (reviewed in [7]). In the bone microenvironment, TGF-β is released from bone during bone resorption and it stimulates breast cancer cells to produce osteolytic factors, such as interleukin 11 (IL-11), that mediate osteolysis by stimulating osteoclast formation and bone resorption activity [8]–[10]. High expression of IL-11 correlates with high histological grade and poor survival in breast cancer [11]. Total systemic blockade of TGF-β signaling pathway by neutralizing antibodies against TGF-β or small molecule inhibitors against the type I TGF-β receptor kinase activity prevents bone metastases in preclinical models [12], [13] but may cause off-target effects because TGF-β has many functions in normal physiology as well as tumor suppressing effects during early stages of breast cancer.

The aim of this study was to elucidate the role of miRNAs in the bone metastatic process of breast cancer and specifically, to identify miRNAs that regulate TGF-β-induced IL-11 expression. We first used array-based miRNA expression profiling of highly bone metastatic variant MDA-MB-231(SA) and parental MDA-MB-231 cells and functional cell-based miRNA overexpression screening to identify miRNAs with a potential role in the TGF-β-induced IL-11 production.

## Results

### MiRNA Expression Profiling of Highly Bone Metastatic MDA-MB-231(SA) and Parental MDA-MB-231 Cells

We have previously demonstrated that despite the remarkably enhanced bone metastatic capability of the MDA-MB-231(SA) variant as compared to the parental MDA-MB-231 cell line in a mouse model of bone metastasis, its genome-wide gene expression and copy number profiles are relatively similar to those of the parental cells [14]. To complement these analyses, we measured the expression of 455 miRNAs in the highly bone metastatic MDA-MB-231(SA) variant and parental MDA-MB-231 cells. We found that 16 (3.5%) of the miRNAs were differentially expressed (>3-fold) between the cell types. Of these, five miRNAs (miR-200b, miR-200a, miR-210, miR-429, and miR-152) were downregulated and eleven (miR-504, miR-196a, miR-142-3p, miR-142-5p, miR-105, miR-517c, miR-363, miR-135b, miR-521, miR-145, and miR-582) upregulated in the highly bone metastatic as compared to the parental MDA-MB-231 cells (Table S1).

### Identification of miRNAs that Regulate IL-11 Production

**Figure 1:**
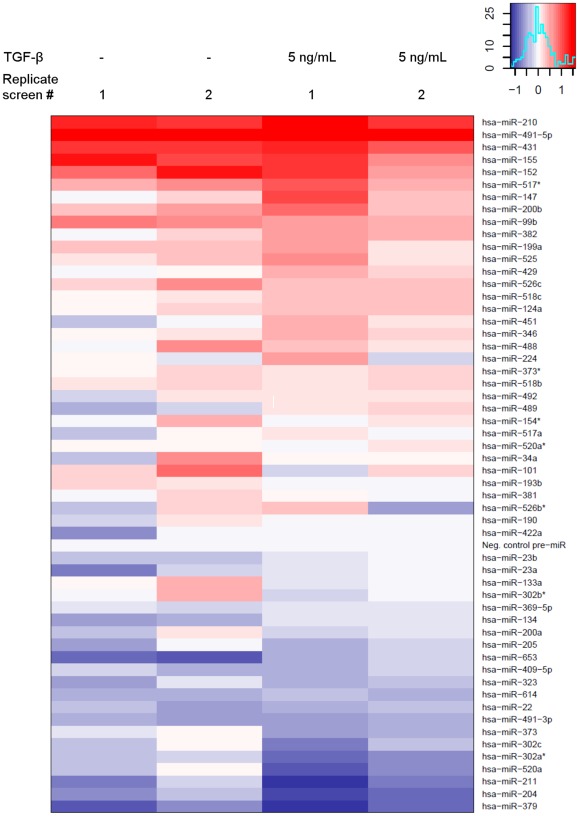
Effects of 55 miRNA precursors and a negative control on IL-11 secretion in MDA-MB-231(SA) cells. Cells were transfected with miRNA precursors in 96-well plates. Medium was changed 24 hours after transfection (+/− TGF-β), and IL-11 concentration in the conditioned medium was measured 24 hours later. IL-11 concentration was normalized to the number of viable cells in the well. The scale of reduction (blue) or increase (red) in IL-11 concentration in the conditioned medium is represented in the color graph in the upper right hand corner of the figure.

**Figure 2:**
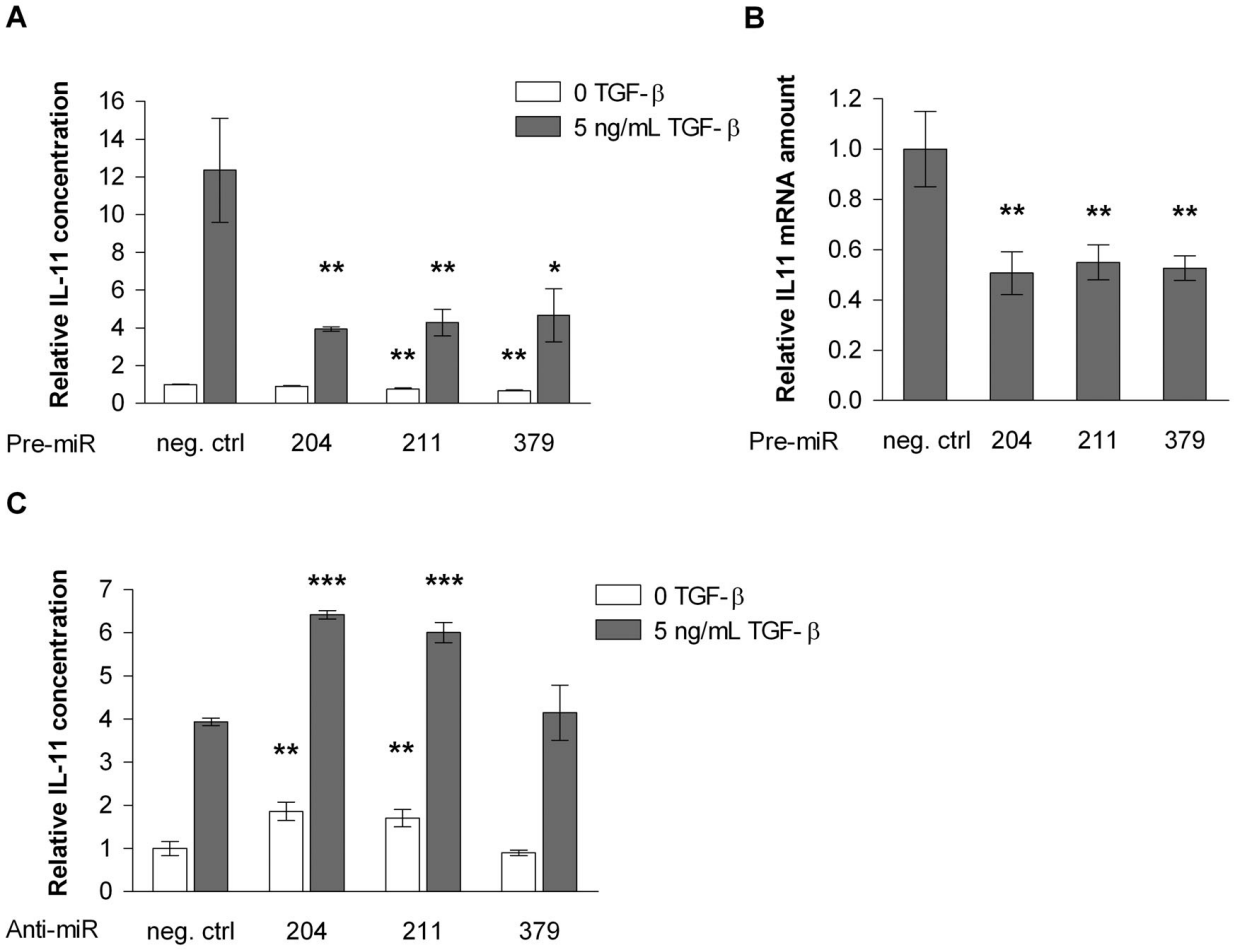
The effects of miR-204, -211 and -379 on IL-11 secretion and IL11 mRNA expression. A. IL-11 concentration in the conditioned medium of MDA-MB-231(SA) cells overexpressing miR-204, -211, or -379 (n = 3). B. *IL11* mRNA expression changes in MDA-MB-231(SA) cells overexpressing miR-204, -211, or -379 (n = 3). C. IL-11 concentration in the conditioned medium of parental MDA-MB-231 cells whose miR-204, -211, or -379 expression was inhibited by specific Anti-miR inhibitors (n = 3). * p<0.05, ** p<0.01, *** p<0.001, as compared to the negative control Pre-miR or Anti-miR.

One of the cell biological properties of the highly metastatic MDA-MB-231(SA) cells is that their TGF-β-induced IL-11 expression is highly elevated as compared to the parental MDA-MB-231 cells [15]. We hypothesized that one or several miRNAs might be responsible for the increased IL-11 production. In order to test this, we performed overexpression screens with miRNA precursors in MDA-MB-231(SA) cells and measured the IL-11 secretion levels in the absence and presence of added TGF-β in the medium. We chose to screen for miRNAs that were downregulated in the MDA-MB-231(SA) cells and the miRNAs that were predicted to target *IL11* according to the miRanda, PicTar, or TargetScan prediction algorithm. This combined selection resulted in 55 miRNAs. Analysis of the replicate screens revealed that the miRNAs >3-fold downregulated in the MDA-MB-231(SA) cells did not have a clear inhibitory effect on IL-11 secretion. Instead, miR-204, -211, and -379 were the most potent inhibitors of IL-11 secretion (Figure 1) and were selected for further studies. MiR-204, -211, and -379 decreased both IL-11 secretion (Figure 2A) and *IL11* mRNA levels (Figure 2B). Furthermore, inhibition of miR-204 and miR-211 by specific Anti-miR inhibitors increased IL-11 secretion in the parental MDA-MB-231 cells (Figure 2C).

### MiR-204, -211, and -379 Bind to the *IL11* 3′ UTR

**Figure 3:**
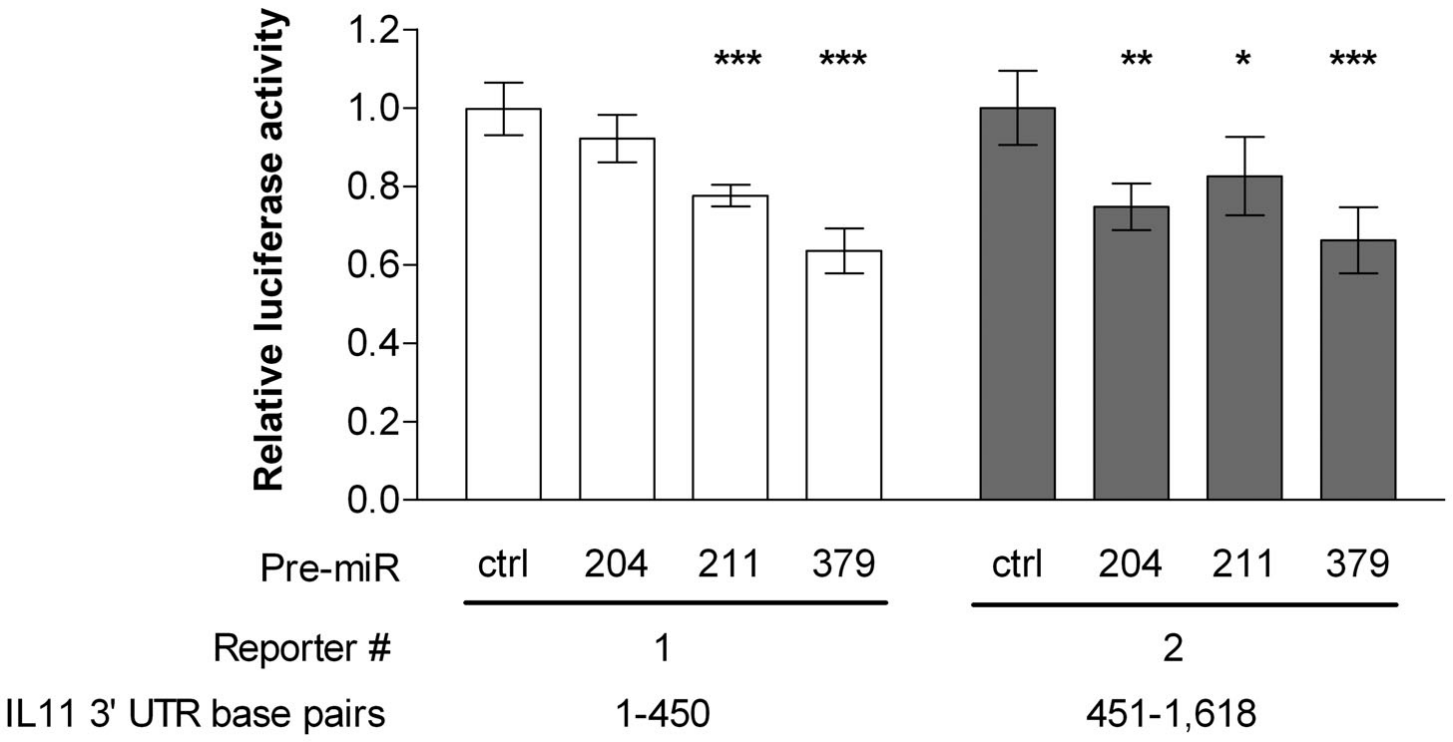
*IL11* 3′ UTR luciferase assay. Luciferase reporter constructs, each containing one fragment of the *IL11* 3′ UTR, were co-transfected with a Renilla luciferase construct and the miRNA precursors into MDA-MB-231(SA) cells (n = 5), and luciferase activity was measured 28 hours later. * p<0.05, ** p<0.01, *** p<0.001, as compared to the negative control Pre-miR.

We wanted to see whether the inhibitory effect of miR-204, -211, and -379 on IL-11 expression was due to direct targeting, i.e. binding of miR-204, -211, and -379 to *IL11* mRNA. First, we used six different miRNA target prediction algorithms (miRanda, PicTar, TargetScan, DIANA-microT, miRDB, and PITA) to investigate in detail the potential of miR-204, -211, and -379 binding to the *IL11* 3′ untranslated region (UTR). Four algorithms, miRanda, DIANA-microT, miRDB, and PITA all predicted two binding sites in the *IL11* 3′ UTR for miR-204 and -211 (Table S2). These two miRNAs share the same seed sequence and only differ in two nucleotides on positions 17 and 18. MiRanda, PicTar, TargetScan, DIANA-microT, or miRDB did not predict binding of miR-379 to the *IL11* 3′ UTR but according to the PITA tool, there are seventeen 6-mer seed sites and one 7- mer seed site for miR-379 in the *IL11* 3′ UTR (Table S2). Subsequently, we performed luciferase reporter assays to determine whether miR-204, -211, or -379 inhibit the expression of the luciferase reporter gene by binding to the *IL11* 3′ UTR sequence. Binding to the 3′ UTR was assayed in two parts: base pairs 1–450 and 451–1,618. A luciferase reporter construct containing one of the two fragments was transfected into MDA-MB-231(SA) cells together with an miRNA precursor and a Renilla luciferase control plasmid. When compared to the negative control Pre-miR, miR-204 inhibited the reporter activity of the construct containing the 451–1,618 end of the UTR. MiR-211 and -379 inhibited the reporter activity of both of the constructs, indicating that they bind to at least two sites in the *IL11* 3′ UTR (Figure 3).

### Additional Gene Expression Changes Induced by miR-204 and miR-379 in MDA-MB-231(SA) Cells

**Figure 4:**
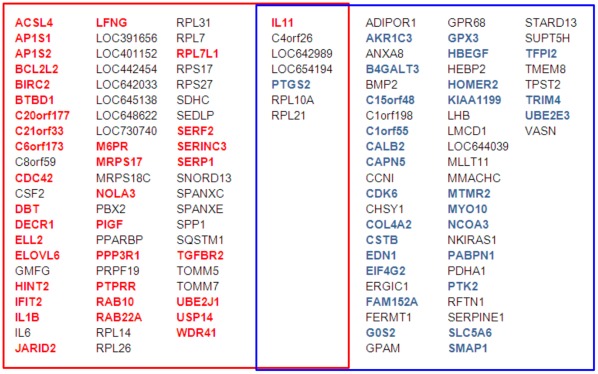
Genes over 1.5-fold downregulated in response to miR-204 and/or -379 versus negative control Pre-miR. RNA samples for genome-wide gene expression analysis were collected 24 hours after MDA-MB-231(SA) cells had been transfected with miRNA precursors. The predicted targets of miR-204 are typed in red and the predicted targets of miR-379 in blue. Expression fold changes, gene names, and more detailed information about the target predictions are listed in the Tables S3 and S4.

Because of the multi-gene regulatory capacity of miRNAs, it is likely that miR-204, -211, and -379 downregulate the expression of *IL11* through additional mechanisms beyond direct targeting. In order to gain a broader understanding of the regulatory functions of these miRNAs, we performed genome-wide gene expression analysis of MDA-MB-231(SA) cells that had been transfected with miR-204 or -379 precursors. MiR-211 was not included in the analysis because it shares the same seed sequence with miR-204. Because we wanted to analyze the primary effects of these miRNAs and not the secondary effects caused by *IL11* silencing, the samples for gene expression analysis were collected already 24 hours after transfection. Furthermore, cells transfected with *IL11* siRNA were also profiled. Using 1.5-fold difference to the negative control siRNA or Pre-miR as a cut-off, the only common gene between the *IL11* siRNA, Pre-miR-204, and Pre-miR-379-treated cells was *IL11*. In addition, cyclin-dependent kinase 6 (CDK6) was downregulated by both miR-379 and *IL11* siRNA. There was an overlap of 15 genes between the genes over 1.5-fold differentially expressed in response to miR-204 and -379. In addition to *IL11*, prostaglandin-endoperoxide synthase 2 (*PTGS2*, also known as COX-2), ribosomal protein L10a (RPL10A), ribosomal protein L21 (RPL21), C4orf26, LOC642989, and LOC654194 were downregulated over 1.5-fold by both miR-204 and -379. Interestingly, miR-204 downregulated the expression of interleukin 1 beta (*IL1B*), interleukin 6 (*IL6*), and osteopontin (*SPP1*). Furthermore, a number of genes that have been reported as direct targets of miR-204/211 [16]–[20], *AP1S2*, *SERP1*, *M6PR*, *RAB22A*, *BIRC2*, *BCL2L2*, and *TGFBR2*, were downregulated over 1.5-fold by miR-204. Of note, miR-379 downregulated the expression of plasminogen activator inhibitor type 1 (*SERPINE1*, also known as *PAI-1*), which is a TGF-β-responsive gene [21], as well as endothelin 1 (*EDN1*, also known as *ET-1*) and bone morphogenetic protein 2 (*BMP2*) (Figure 4, Tables S3 and S4).

The genes that were over 1.5-fold downregulated in response to miR-204 or -379 versus negative control Pre-miR were compared against the gene signatures in the Molecular Signatures Database (MSigDB). Among the MSigDB microRNA target sets, miR-204 showed a significant overlap exclusively with the MSigDB set of genes containing a 3′ UTR binding motif for miR-204 (p = 8.13×10^−4^) (Table S5). Respectively, the genes downregulated in response to miR-379 correlated significantly with the MSigDB miR-379 gene set only (p = 6.13×10^−4^). In addition, there was a significant correlation between the genes downregulated by miR-379 and a set of genes upregulated in basal subtype of breast cancer samples (p = 2.82×10^−5^) and a set of genes downregulated in luminal-like breast cancer cell lines compared to the mesenchymal-like ones (p = 1.06×10^−7^) (Table S6).

### MiR-379 Inhibits Smad Signaling

**Figure 5:**
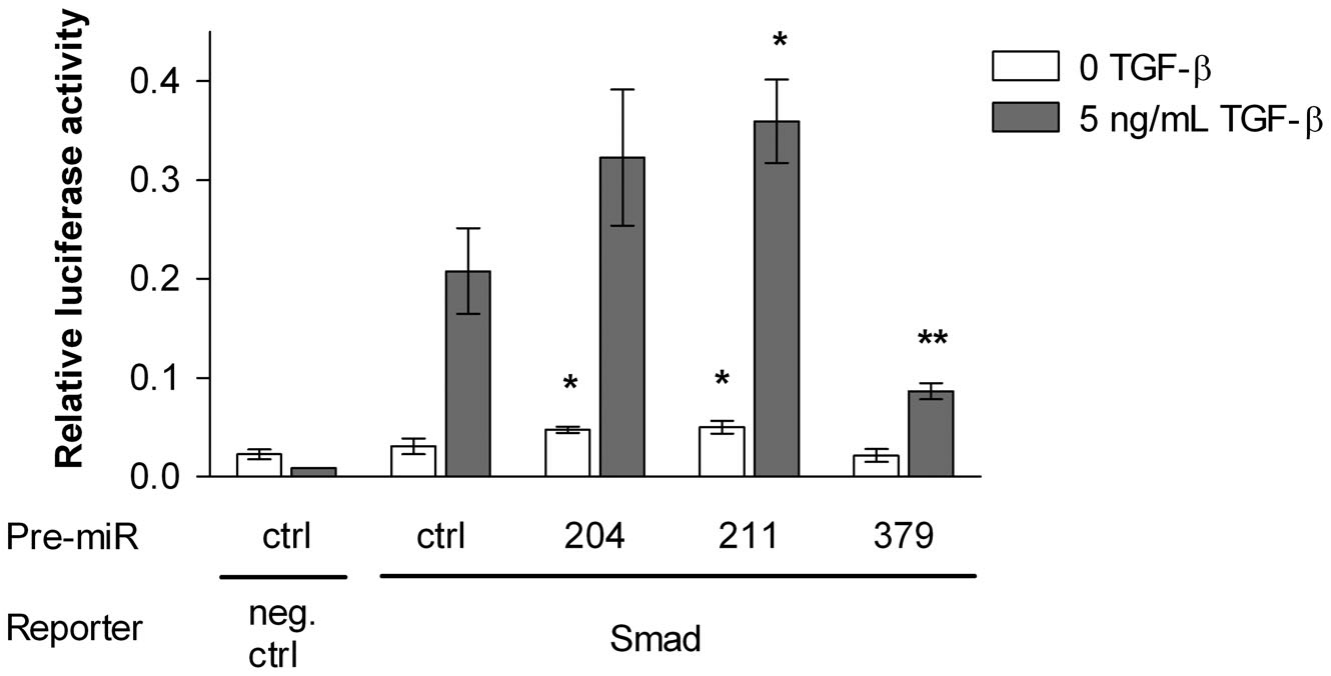
Effects of miR-204, -211, and -379 on Smad signaling. Smad signaling was quantified using a luciferase construct which encodes the firefly luciferase reporter gene under the control of a minimal (m)CMV promoter and tandem repeats of the Smad transcriptional response element. The luciferase reporter construct and miRNA precursors were co-transfected into MDA-MB-231(SA) cells (n = 3), and the medium was replaced with serum-free medium 16 hours after transfection. TGF-β was added 8 hours later. Activity of the firefly luciferase reporter and Renilla luciferase was measured after 16 hour TGF-β induction. * p<0.05, ** p<0.01, as compared to the Pre-miR negative control and Smad reporter-transfected cells.

Given the above results on the inhibitory effects of miR-204 and -379 on the expression of not only *IL11* but also other genes that have been shown to be regulated by TGF-β, such as *PTGS2* (miR-204 and -379) and *SERPINE1* (miR-379), we next studied whether miR-204, -211, or -379 affected Smad signaling. Binding of TGF-β to the TGF-β type II receptor induces the TGF-β receptor type I kinase activity and leads to the phosphorylation of Smad2 and Smad3. Phosphorylated Smads form heteromeric complexes with Smad4, and the Smad complex translocates into the nucleus and interacts with transcriptional activators and repressors to regulate target gene expression. We measured the effects of miR-204, -211, and -379 on Smad2/3/4-mediated transcriptional activity using a luciferase reporter construct containing a functional Smad binding site. Surprisingly, miR-211 (as well as miR-204 but not significantly) increased the TGF-β-induced luciferase signal, indicating an increase in Smad binding to its response element. MiR-379 in turn inhibited the Smad-mediated transcriptional activity as shown by a marked reduction in the luciferase signal (Figure 5). We also measured the effects of Anti-miR-204 and -379 on Smad signaling in the MDA-MB-231(SA) as well as in the parental MDA-MB-231 cells. Inhibition of miR-204 led to a decrease in the TGF-β-induced luciferase signal in both cell types. Anti-miR-379 increased the signal in the MDA-MB-231(SA) cells but did not show any significant effect in the parental MDA-MB-231 cells (Figure S1).

## Discussion

In our comparison of the miRNA expression profiles between the highly bone metastatic MDA-MB-231(SA) variant and the parental MDA-MB-231 cell line, we found 16 miRNAs that were differentially expressed by more than 3-fold. The miR-200 family was strongly represented: three of the five family members, miR-200b, -200a, and -429, were among the four most downregulated miRNAs in the highly metastatic MDA-MB-231(SA) cells. The role of these miRNAs in EMT has previously been reported by Gregory et al. [2]. Interestingly, miR-210, whose high expression has previously been associated with increased likelihood of distant metastasis [22] and poor clinical outcome [23] in breast cancer, was 6.5-fold downregulated in the MDA-MB-231(SA) cells.

Because of the highly increased IL-11 production in MDA-MB-231(SA) cells in response to TGF-β, we chose to screen for miRNAs whose overexpression inhibits IL-11 production in MDA-MB-231(SA) cells. We identified miR-204, -211, and -379 as the strongest modulators of IL-11 production. We demonstrated that miR-204, -211, and -379 reduced not only IL-11 secretion but *IL11* mRNA levels as well. Furthermore, our results from the luciferase reporter assays indicated that miR-204 and -211 bind to the *IL11* 3′ UTR which is in concordance with the predictions of several different bioinformatic algorithms that predicted *IL11* as a direct target for miR-204 and -211. To our knowledge, miR-211 or -379 have not been previously linked to IL-11 production or breast cancer metastasis. In regard to miR-204, our finding is in line with a previous report showing reduced expression of IL-11 at protein level in miR-204-transfected human trabecular meshwork cells [19].

According to miRNA target prediction tools, binding of miR-379 to the *IL11* 3′ UTR seemed unlikely. However, our laboratory experiments showed that also miR-379 binds to the *IL11* 3′ UTR. This inconsistency might be due to the fact that common prediction algorithms mainly focus on seeds of 7–8 base pairs, and there is only one 7-base pair seed match and no 8-base pair matches for miR-379 in the *IL11* 3′ UTR. It was recently reported that up to 67% of functional miRNA binding sites are based on 6-mer seeds [24]. Recent high-throughput analyses of miRNA-mRNA pairing also suggest that 25–45% of binding sites lack a perfect seed match [25], [26], and there is an increasing list of validated binding sites lacking a canonical seed [27]–[30]. Together with these previously published evidence, our finding on the miR-379 binding to the *IL11* 3′ UTR indicates that bioinformatic prediction of miRNA targeting is lacking sensitivity, reflecting our insufficient knowledge of miRNA binding patterns.

Inhibition of miR-204 or -211 in the parental MDA-MB-231 cells led to increased IL-11 secretion, suggesting that these miRNAs could be one underlying reason for the low levels of secreted IL-11 in these cells as compared to the highly metastatic variant. Inhibition of miR-379, in turn, did not significantly affect IL-11 secretion in the parental cells. This indicates that apart from direct targeting of *IL11* mRNA, miR-379 and miR-204/−211 work through different mechanisms and that there is a regulatory mechanism keeping the IL-11 levels down in the MDA-MB-231 cells which is not significantly affected by the inhibition of miR-379. In addition, our results from the gene expression profiling of miR-204 and -379-transfected MDA-MB-231(SA) cells showed that the overlap between the gene expression changes caused by these miRNAs was low, suggesting that except for downregulating *IL11* expression, miR-204 and -379 largely engage in distinct regulatory pathways. However, both miR-204 and -379 downregulated the expression of *PTGS2*. Based on previous publications, TGF-β stimulates *PTGS2* expression in breast cancer cells [31] and silencing of *PTGS2* decreases *IL11* expression in MDA-MB-231 cells [32], [33]. Therefore, in addition to direct targeting, the inhibitory effects of miR-204 and -379 on IL-11 expression might in part be an indirect consequence of reduced *PTGS2* activity. In line with a previous finding by Wang et al. [16], we also saw downregulation of *TGFBR2* in response to miR-204. This is an additional mechanism likely to contribute to the attenuated TGF-β induction of *IL11* in miR-204-transfected cells. Furthermore, miR-204 downregulated *CDC42*, which has been implicated in non-Smad TGF-β signaling [34], [35], and *IL6*, a transcriptional target of TGF-β [36]. MiR-379, in turn, downregulated the expression of *BMP2* and a TGF-β target gene *SERPINE1*, both of which have been implicated in the development of breast cancer bone metastases [37]–[40].

We further delineated the TGF-β related regulatory mechanisms of miR-204 and -379 using a Smad reporter assay and demonstrated that miR-379 inhibited the TGF-β-induced binding of the Smad complex to its response element, indicating an additional contributing mechanism for the *IL11* inhibition by miR-379. In contrast, miR-211 (as well as miR-204 but not significantly) increased the Smad transcriptional activity. These findings were supported by the opposing effects of specific Anti-miR inhibitors against miR-204 and -379. TGF-β signaling through the Smad pathway is a critical mediator of bone metastasis and regulates the expression of several bone metastasis-relevant growth factors in breast cancer cells. Blockade of Smad-mediated TGF-β signaling by overexpression of Smad7 in breast cancer cells has been shown to reduce bone metastases [41]. In addition, TGF-β signaling induces EMT, whereby epithelial cancer cells acquire a mesenchymal-like phenotype with motile and invasive characteristics. Our finding on the inhibitory effect of miR-379 on Smad signaling is consistent with our gene expression profiling analysis showing a highly significant correlation between the genes downregulated by miR-379 and two previously published gene sets: genes upregulated in basal subtype of clinical breast cancer samples and genes downregulated in luminal-like breast cancer cell lines as compared to the mesenchymal-like ones. This underlines the functional relevance of the gene signature induced by miR-379 and also suggests associations of clinical relevance in regard to EMT and breast cancer subtypes.

In conclusion, our studies bring several novel insights into the miRNA-driven mechanisms of bone metastasis. First, low expression of miR-200a, -200b, and -429 in highly bone metastatic MDA-MB-231(SA) cells is in line with the previously reported metastasis-regulating functions of these miRNAs. Second, miR-204, -211, and -379 directly downregulate a key pathogenetic process in breast cancer metastasis, the TGF-β-induced expression of *IL11*. MiR-379 also inhibits TGF-β signaling through Smad2/3/4 transcription factors. Finally, miR-204 and -379 downregulate the expression of several metastasis-relevant genes, and miR-379 downregulates genes typically highly expressed in the basal breast cancer subtype and genes downregulated in luminal-like breast cancer cell lines. Thus, our results suggest that these miRNAs may be key cellular mediators of the metastatic process in breast cancer *in vitro*, with potential clinical relevance.

## Materials and Methods

### Cell lines and Culture Conditions

The parental MDA-MB-231 cell line was obtained from the American Type Culture Collection (ATCC). MDA-MB-231(SA) cells were spontaneously derived from these cells during *in vitro* culture and comprehensively characterized by comparative genomic hybridization and genome-wide gene expression profiling by us as described in [14]. Cells were maintained in low-glucose DMEM medium with 10% inactivated fetal bovine serum (FBS), 1% nonessential amino acids, 100 U/mL penicillin and 100 µg/mL streptomycin in an incubator with a humidified atmosphere of 95% air and 5% CO_2_ at 37°C.

### MiRNA Expression Analysis

RNA was isolated from MDA-MB-231(SA) and parental MDA-MB-231 cells using the mirVana miRNA Kit (Applied Biosystems). MiRNA microarray analyses were done by Agilent Technologies Inc. using the Agilent microarray platform v1, which contained probes for 455 human miRNAs. The data were normalized by variance stabilization [42].

### IL-11 and Cell Viability Assays

Cells were transfected with miRNA precursors (Pre-miR, Ambion) or miRNA inhibitors (Anti-miR, Ambion) at a final concentration of 22 nM in 384-well plates or 20 nM in 96-well plates. SiLentFect (Bio-Rad) was incubated with OptiMEM medium for 10 minutes after which the mixture was added to the wells containing the preprinted Pre-miRs or Anti-miRs. Cells were treated with trypsin inhibitor (Sigma-Aldrich) and plated for transfection in low-glucose DMEM medium with 1% nonessential amino acids, 0.5% BSA, and 100 U/mL penicillin and 100 µg/mL streptomycin. 24 hours after transfection, medium was changed and TGF-β was added to the final concentration of 5 ng/mL. Medium samples were collected 48 hours after transfection. IL-11 concentration in the conditioned medium was measured using the DuoSet ELISA Development System for human IL-11 (R&D Systems) according to the manufacturer’s instructions. IL-11 concentration was normalized to the number of viable cells in the well which was measured using the CellTiter-Blue assay (Promega Corporation, Madison, WI, USA) according to the manufacturer’s instructions.

### Analysis of Gene Expression by Quantitative Real-time RT-PCR

RNA isolation, reverse transcription, and TaqMan analyses were done as previously described [14]. The primers and probes are listed in the Table S7.

### Luciferase Reporter Assays

The luciferase assays were done in 96-well plates. Binding to the *IL11* 3′ UTR sequence was assayed in two parts: base pairs 1–450 (1) and 451–1,618 (2), and two luciferase reporter constructs, each containing one of these fragments. These constructs were ordered from SwitchGear Genomics (Menlo Park, CA, USA). One of the *IL11* 3′ UTR luciferase reporter constructs (70 ng) along with miRNA precursors (30 nM) and Renilla luciferase plasmid (20 ng) as a control were co-transfected into MDA-MB-231(SA) cells using 0.5 µL Lipofectamine 2000 (Invitrogen) according to the manufacturer’s instructions. Luciferase levels were measured 28 hours after transfection, using the Dual-Glo Luciferase Assay System (Promega Corporation) according to the manufacturer’s instructions. The background signal from the untransfected cells was subtracted, and the firefly reporter values were divided with the respective Renilla values. Smad signaling was quantified using the Cignal Smad Reporter Assay Kit (SABiosciences, Fredrick, MD, USA). MiRNA precursors (20 nM) or Anti-miR inhibitors (50 nM) along with 100 ng luciferase reporter construct were co-transfected into the cells using 0.5 µL Lipofectamine 2000. Medium was replaced with serum-free medium 6–16 hours after transfection, and 5 ng/ml TGF-β was added 8–17 hours later. Activity of the firefly luciferase reporter and Renilla luciferase was measured after 8–16 hour TGF-β induction using the Dual-Glo Luciferase Assay System as described above for the UTR reporter assay. The exact time points are indicated in the figure legends.

### Gene Expression Profiling

MDA-MB-231(SA) cells were transfected with miRNA precursors, *IL11* siRNA (catalog number SI00013475, Qiagen), or negative control siRNA (catalog number 1027281, Qiagen) at a final concentration of 20 nM as described for the IL-11 assay above and plated in low-glucose DMEM medium with 1% nonessential amino acids, 0.5% BSA, 100 U/mL penicillin and 100 µg/mL streptomycin, and 5 ng/mL TGF-β in 6-well plates. Total RNA was extracted from the cells 24 hours after transfection. The RNA was purified using the mirVana kit according to the manufacturer’s instructions. Purified total RNA (300 ng) was amplified with the Illumina RNA TotalPrep Amplification kit (Ambion) and biotinylated. The RNA/cRNA concentrations were measured with Nanodrop ND-1000 before and after the amplifications, and RNA/cRNA quality was controlled by BioRad’s Experion electrophoresis station. The biotin labeled cRNA (0.75 µg) was hybridized to Illumina Sentrix HumanHT-12 Expression BeadChips, version 3, at 58°C for 18 hours according to the Illumina Whole-Genome Gene Expression Direct Hybridization protocol, revision A. The hybridization was detected with 1 µg/ml Cyanine3-streptavidine (GE Healthcare Limited, UK). The arrays were scanned with Illumina BeadArray Reader, BeadScan software version 3.5. Data analysis and visualization was done using standard and custom algorithms implemented in the R/BioConductor framework for statistical computing [43], [44]. Raw data was transformed using the variance-stabilizing transformation method described by Lin et al. [45] and implemented in the ‘lumi’ package [46]. A linear model was fit to the data, and differential gene expression was assessed by computing empirical Bayes statistics as implemented in the ‘limma’ package [47]. The data were deposited in the ArrayExpress database (http://www.ebi.ac.uk/arrayexpress/) and are accessible under the accession number E-TABM-1220.

### MiRNA Target Prediction

MiRNA precursors were selected for the cell-based screen using the following prediction algorithms: miRanda [48], PicTar [49], and TargetScan version 4.0 [50]. The subsequent analyses of the potential binding sites for miR-204, -211, and -379 in the 3′ UTR of *IL11* and the genes that were downregulated in response to miR-204 or -379 were done using the miRNA target prediction algorithms miRanda (microRNA.org August 2010 release), PicTar, TargetScan version 5.2, DIANA-microT version 3.0 [51], [52], miRDB version 3.0 [53], [54], and PITA version 6 [55].

### Statistical Analysis

The results are reported as mean ± SD. Statistical significance of the results were analyzed using an unpaired Student’s *t* test.

## Supporting Information

Figure S1 **Effects of Anti-miR-204 and -379 on Smad signaling in MDA-MB-231(SA) (A) and parental MDA-MB-231 (B) cells.** Smad signaling was quantified using a luciferase construct which encodes the firefly luciferase reporter gene under the control of a minimal (m)CMV promoter and tandem repeats of the Smad transcriptional response element. The luciferase reporter construct and the Anti-miR were co-transfected into MDA-MB-231(SA) (n = 4) or MDA-MB-231 cells (n = 3), and the medium was replaced with serum-free medium 6 hours after transfection. TGF-β was added 17 hours later. Activity of the firefly luciferase reporter and Renilla luciferase was measured after 8 hour TGF-β induction. * p<0.05, ** p<0.01, as compared to the Anti-miR negative control and Smad reporter-transfected cells.
(TIF)

Table S1 Expression of 455 miRNAs in highly metastatic MDA-MB-231(SA) and parental MDA-MB-231 cells.
(XLS)

Table S2 Predicted miRNA seed sequence binding sites in the *IL11* 3' UTR. The number of mismatches and G:U wobble pairs are given in the parentheses.
(XLS)

Table S3 Genes over 1.5-fold under- or overexpressed in response to miR-204.
(XLS)

Table S4 Genes over 1.5-fold under- or overexpressed in response to miR-379.
(XLS)

Table S5 Overlap between the genes over 1.5-fold downregulated in response to miR-204 in MDA-MB-231(SA) cells and gene sets in the Molecular Signatures Database (MSigDB). The most significantly overlapping gene sets are shown.
(XLS)

Table S6 Overlap between the genes over 1.5-fold downregulated in response to miR-379 in MDA-MB-231(SA) cells and gene sets in the Molecular Signatures Database (MSigDB). The most significantly overlapping gene sets are shown.
(XLS)

Table S7 Primers and probes used for quantitative RT-PCR.
(XLS)
